# Association of Early Progression Independent of Relapse Activity With Long-term Disability After a First Demyelinating Event in Multiple Sclerosis

**DOI:** 10.1001/jamaneurol.2022.4655

**Published:** 2022-12-19

**Authors:** Carmen Tur, Pere Carbonell-Mirabent, Álvaro Cobo-Calvo, Susana Otero-Romero, Georgina Arrambide, Luciana Midaglia, Joaquín Castilló, Ángela Vidal-Jordana, Breogán Rodríguez-Acevedo, Ana Zabalza, Ingrid Galán, Carlos Nos, Annalaura Salerno, Cristina Auger, Deborah Pareto, Manuel Comabella, Jordi Río, Jaume Sastre-Garriga, Àlex Rovira, Mar Tintoré, Xavier Montalban

**Affiliations:** 1 Multiple Sclerosis Centre of Catalonia, Department of Neurology/Neuroimmunology, Hospital Universitari Vall d’Hebron, Universitat Autònoma de Barcelona, Barcelona, Spain; 2 Section of Neuroradiology, Department of Radiology, Vall d’Hebron University Hospital, Spain. Universitat Autònoma de Barcelona, Barcelona, Spain

## Abstract

**Importance:**

Progression independent of relapse activity (PIRA) is the main event responsible for irreversible disability accumulation in relapsing multiple sclerosis (MS).

**Objective:**

To investigate clinical and neuroimaging predictors of PIRA at the time of the first demyelinating attack and factors associated with long-term clinical outcomes of people who present with PIRA.

**Design, Setting, and Participants:**

This cohort study, conducted from January 1, 1994, to July 31, 2021, included patients with a first demyelinating attack from multiple sclerosis; patients were recruited from 1 study center in Spain. Patients were excluded if they refused to participate, had alternative diagnoses, did not meet protocol requirements, had inconsistent demographic information, or had less than 3 clinical assessments.

**Exposures:**

Exposures included (1) clinical and neuroimaging features at the first demyelinating attack and (2) presenting PIRA, ie, confirmed disability accumulation (CDA) in a free-relapse period at any time after symptom onset, within (vs after) the first 5 years of the disease (ie, early/late PIRA), and in the presence (vs absence) of new T2 lesions in the previous 2 years (ie, active/nonactive PIRA).

**Main Outcomes and Measures:**

Expanded Disability Status Scale (EDSS) yearly increase rates since the first attack and adjusted hazard ratios (HRs) for predictors of time to PIRA and time to EDSS 6.0.

**Results:**

Of the 1128 patients (mean [SD] age, 32.1 [8.3] years; 781 female individuals [69.2%]) included in the study, 277 (25%) developed 1 or more PIRA events at a median (IQR) follow-up time of 7.2 (4.6-12.4) years (for first PIRA). Of all patients with PIRA, 86 of 277 (31%) developed early PIRA, and 73 of 144 (51%) developed active PIRA. Patients with PIRA were slightly older, had more brain lesions, and were more likely to have oligoclonal bands than those without PIRA. Older age at the first attack was the only predictor of PIRA (HR, 1.43; 95% CI, 1.23-1.65; *P* < .001 for each older decade). Patients with PIRA had steeper EDSS yearly increase rates (0.18; 95% CI, 0.16-0.20 vs 0.04; 95% CI, 0.02-0.05; *P* < .001) and an 8-fold greater risk of reaching EDSS 6.0 (HR, 7.93; 95% CI, 2.25-27.96; *P* = .001) than those without PIRA. Early PIRA had steeper EDSS yearly increase rates than late PIRA (0.31; 95% CI, 0.26-0.35 vs 0.13; 95% CI, 0.10-0.16; *P* < .001) and a 26-fold greater risk of reaching EDSS 6.0 from the first attack (HR, 26.21; 95% CI, 2.26-303.95; *P* = .009).

**Conclusions and Relevance:**

Results of this cohort study suggest that for patients with multiple sclerosis, presenting with PIRA after a first demyelinating event was not uncommon and suggests an unfavorable long-term prognosis, especially if it occurs early in the disease course.

## Key Points

**Question:**

What are the long-term outcomes of patients developing progression independent of relapse activity (PIRA) after a first demyelinating event in multiple sclerosis?

**Findings:**

In this longitudinal cohort study including 1128 patients with a first demyelinating event in multiple sclerosis, presenting with PIRA was associated with significantly shorter times to developing severe disability compared with not presenting with PIRA. Patients presenting with PIRA within the first 5 years of multiple sclerosis had a significantly 26-fold greater risk of developing severe disability than patients whose first PIRA appeared late in the disease.

**Meaning:**

Results suggest that presenting with PIRA after a first demyelinating event in multiple sclerosis is an ominous prognosis, especially if it occurs early in the disease course.

## Teaser

This cohort study investigates the long-term outcomes of patients who develop progression independent of relapse activity after a first demyelinating event in multiple sclerosis.

## Introduction

In multiple sclerosis (MS), the irreversible accumulation of disability may occur at any stage of the disease^1,2,3,4^ and through 2 main mechanisms: relapse-associated worsening (RAW) and progression independent of relapse activity (PIRA).^4^ Nonetheless, PIRA, associated with a predominant underlying neurodegenerative component,^2,3,4,5^ seems to be the most important mechanism even in patients with no formal diagnosis of secondary progressive MS.^3,4^

PIRA has been studied in patients with very early MS, including patients after a first demyelinating attack of the central nervous system^6^ and patients with established MS.^2,4^ However, the clinical and neuroimaging predictors of PIRA at the time of the first demyelinating event have not yet, to our knowledge, been investigated. Additionally, the long-term disability outcomes of patients who present with PIRA are still largely unknown. Considering that PIRA may be understood as the first clinical sign of progression in a relapsing-remitting context, it is important to know whether patients who develop their first PIRA event very early in the disease course show a particularly unfavorable prognosis. Furthermore, the association of PIRA with brain inflammatory activity is still unclear.^2,4^

With this longitudinal study of a uniquely large cohort of patients with a first demyelinating event,^7,8^ we aimed to estimate the risk of PIRA after symptom onset and investigate its potential clinical and magnetic resonance imaging (MRI) predictors at the time of such first event. We also aimed to evaluate the long-term evolution of those patients with PIRA and understand the potential association between the timing of the first PIRA event or the presence of recent inflammatory activity before PIRA and the long-term disability outcomes.

## Methods

### Study Design and Participants

This was a retrospective analysis of data from patients prospectively included in the deeply phenotyped Barcelona cohort of patients with a first demyelinating attack from the Multiple Sclerosis Center of Catalonia^7,8,9^ between January 1, 1994, and July 31, 2021. The study protocol was evaluated by the ethics committee of Vall d’Hebron Hospital. The cohort included patients younger than 50 years who experienced a first demyelinating event of the central nervous system that could not be attributed to other diseases. Patients were assessed at the Multiple Sclerosis Center of Catalonia within 3 months of the first demyelinating attack.^7^ For the current study, only those patients with at least 3 Expanded Disability Status Scale (EDSS) assessments were included. All patients provided written informed consent. This study followed the Strengthening the Reporting of Observational Studies in Epidemiology (STROBE) reporting guidelines.

### Demographic, Clinical, and Paraclinical Data

The included information consisted of demographics (sex, age at first demyelinating attack) and clinical data (date and topography of the first demyelinating event, presence and dates of relapses [recorded at each visit], disability status according to the EDSS,^10^ and disease-modifying treatment [DMT] sequences [DMT onset and stop dates]). EDSS scores were obtained within 3 months after the first demyelinating attack and then (at least) annually. Also collected were paraclinical data, ie, the presence of oligoclonal bands (OBs) in the cerebrospinal fluid (CSF) and serum at the first attack, which were tested on agarose gel isoelectric focusing combined with immunoblotting^11^ and MRI data of the brain and spinal cord, which included the number and topography of T2 lesions and the presence of contrast-enhancing lesions (CELs) at the first attack and the number of new brain T2 lesions on follow-up MRI scans. Brain MRI scans were performed within the first 5 months after symptom onset, then 12 months afterward, and at least every 5 years thereafter, for all patients. Additionally, patients could undergo a new brain MRI scan when new symptoms were reported or before starting any new treatment.^12^ After 2007, spinal cord MRI scans were performed systematically for all patients at study baseline (ie, within 5 months of the first demyelinating attack), regardless of the topography of the first attack.

### Definition of PIRA and PIRA Subgroups

#### Definition of PIRA and RAW

**Figure 1.**
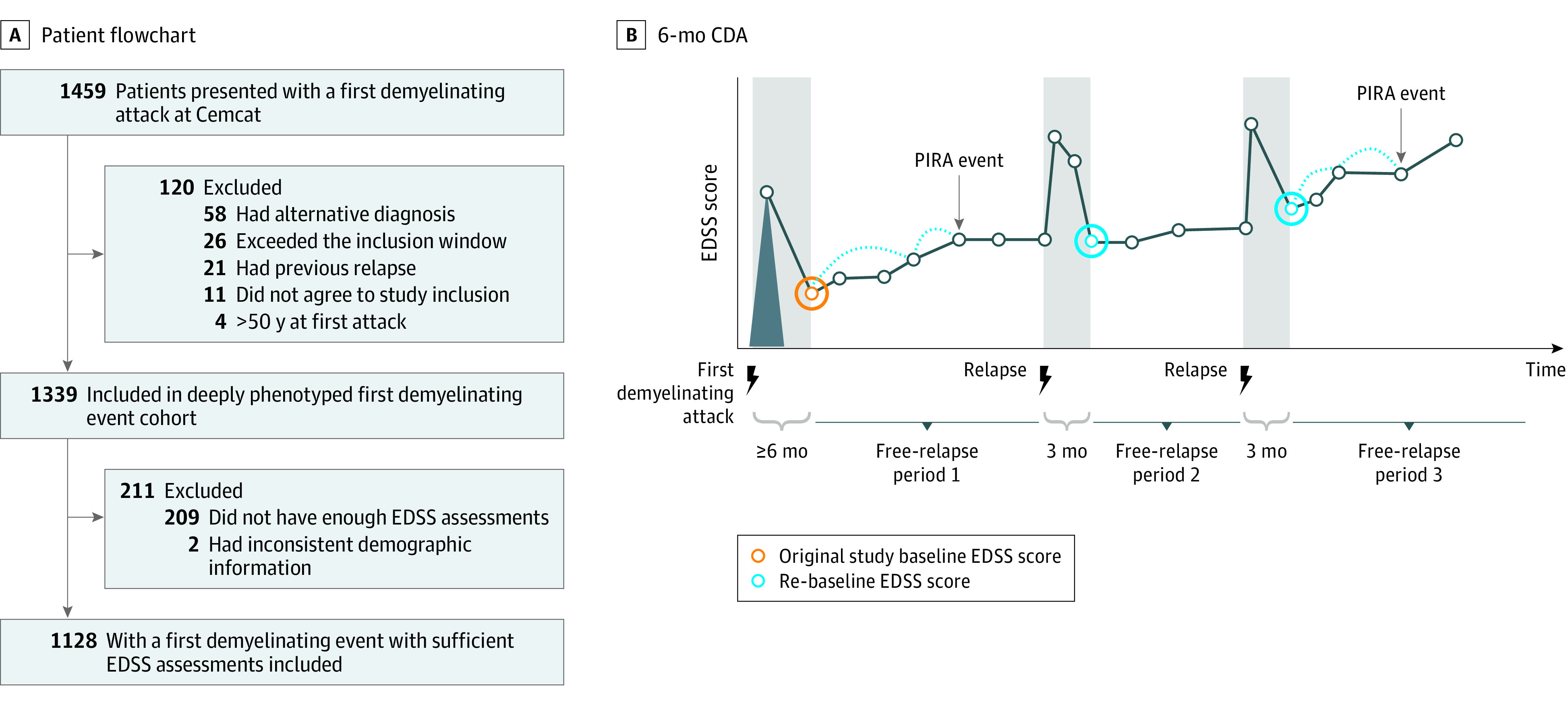
Patient Flowchart and 6-Month Confirmed Disability Accumulation (CDA) A, Patient flowchart from the whole deeply phenotyped cohort until the study cohort, with 1128 patients. B, Display of how the events of 6-month CDA were considered. The periods shaded in gray (6 months after the first attack and 3 months after any other event) represent the periods where any increase in Expanded Disability Status Scale (EDSS) score is considered to be associated with relapses. The time outside of these gray-shaded periods is referred to as periods free of relapses (PFRs). Any CDA within a PFR was considered a progression independent of relapse activity (PIRA) event, whereas any CDA outside a PFR was considered a relapse-associated worsening event. Patients with at least 1 CDA who had at least 1 PIRA event were considered patients with PIRA. Instead, patients with at least 1 CDA who never presented with a PIRA event were called patients with a relapse-associated worsening event. CDA was considered when a minimum EDSS increase was observed: 1.5, 1.0, or 0.5 points, with respect to a baseline/rebaseline EDSS score of 0, 1.0 to 5.0, or greater than 5.0, respectively.

We defined a PIRA event as experiencing confirmed disability accumulation (CDA) in the EDSS scale at 6 months during a period free of relapses (PFRs) (Figure 1). A PFR was the time between 2 consecutive relapses, starting 3 months after a relapse (or 6 months after the first demyelinating event). The first EDSS score obtained at least 6 months after the first attack or 3 months after any other attack was referred to as the baseline EDSS score and rebaseline EDSS score, respectively. We set that no rebaseline EDSS score could be lower than the first recorded (baseline) EDSS score.^4^ CDA was defined as an increase in the EDSS score of 1.5, 1.0, or 0.5 if the baseline/rebaseline EDSS score was, respectively, 0, 1.0 to 5.0, or greater than 5.0. The date of PIRA was the date of the confirmation of the CDA. Any other episodes of CDA that did not qualify for PIRA (ie, which occurred outside the PFR) were considered to be RAW events. Those patients with at least 1 CDA but who did not present with any PIRA event were considered patients with RAW.

#### PIRA Subgroups

All patients with PIRA were classified into early PIRA or late PIRA groups, depending on whether the first PIRA event occurred within the first 5 years since their first attack or afterward, respectively; the choice of a 5-year cutoff is taken from previous longitudinal studies of primary progressive MS, which considered early disease to be disease with a duration less than 5 years.^13,14,15,16^ Patients with PIRA were further classified into active PIRA or nonactive PIRA groups, depending on the presence or absence, respectively, of new T2 lesions observed in the 2 years before developing PIRA. The latter classification was applied on a subcohort of patients with a brain MRI scan available within the 2 years before developing PIRA.

### Statistical Analyses

#### Descriptive Statistics and Baseline Comparisons

We assessed the proportion of patients developing PIRA and compared clinical/paraclinical characteristics across groups (PIRA vs no PIRA, PIRA vs RAW, PIRA subgroup comparisons) at the time of the first demyelinating event using parametric or nonparametric tests as appropriate.

Kaplan-Meier and Cox regression models were built to assess, respectively, the risk of PIRA and the association of baseline (first attack) characteristics (age, sex, topography of first attack, brain and spinal cord lesion number categories, brain CEL number category, and OBs) with such risk. Cox models were also adjusted for (binary) DMT exposure (treated as a time-varying covariate, assuming that treatment exposure started when the first DMT started and finished at the end of follow-up) and for percentage time receiving high-efficacy drugs (out of total follow-up). eTable 1 in the Supplement shows the high-efficacy drugs prescribed to our patients. For all survival models, the proportional hazard assumption was assessed through visual inspection of scaled Schoenfeld residuals and through scaled Schoenfeld residuals test from Stata/SE, version 14.2 (StataCorp).

#### Long-term Clinical Outcomes

Comparisons across groups (ie, PIRA vs no PIRA, PIRA vs RAW, or PIRA subgroups) were made with respect to different longitudinal outcomes: (1) the yearly rates of EDSS increase since the first demyelinating attack and (2) the risk of reaching 6-month confirmed EDSS 6.0 from the first demyelinating attack.

The yearly rates of EDSS increase since the first demyelinating attack were evaluated through linear mixed models. In such models, the EDSS score at each time point was considered as the dependent variable, and time (in years) was the main explanatory variable. Moreover, we included, as a covariate, a quadratic term for time in order to account for a nonlinear behavior of the EDSS increase over time. To assess differences between groups, a binary indicator of group (eg, PIRA/no PIRA) and an interaction term such as time *X* binary group indicator were also included as covariates. Whenever the interaction term was significant, we assumed that the 2 groups differed in terms of EDSS changes over time.

All these models were adjusted for the following first attack–related covariates: age, sex, topography of first attack, brain and spinal cord lesion number categories, brain CEL number category, and OBs. Models were also adjusted for the proportion of time receiving DMTs and the proportion of time receiving high-efficacy DMTs (out of total follow-up in both cases). Mixed models had random intercept (for patient) and random slope (for time), with unstructured covariance structure.

The risk of reaching 6-month confirmed EDSS 6.0 from the first demyelinating attack was evaluated with Kaplan-Meier and Cox regression models, including a binary indicator of group (eg, PIRA/no IRA) as an explanatory variable. Kaplan-Meier estimates were compared across groups through log-rank tests. Cox models were adjusted for first attack–related covariates: age, sex, first attack topography, brain and spinal cord lesion number categories, brain CEL number category, and OBs. Models were also adjusted for DMT exposure: binary time-varying covariate (for any DMT) plus adjustment for the percentage of time receiving high-efficacy drugs (out of total follow-up). Adjusted hazard ratios (HRs) for predictors of time to EDSS 6.0 were obtained.

#### Sensitivity Analyses

In order to understand to what extent our results could be influenced by the presence of a large proportion of patients with a first demyelinating attack who most likely would never progress, we repeated all our analyses with only those patients who fulfilled McDonald 2017 criteria at any time during the follow-up.

Statistical evidence was considered when 2-sided *P* values were <.05. Statistical analyses were performed with R Core team, version 3.6.0 (R Foundation Statistical Computing), and Stata/SE, version 14.2 (StataCorp).

## Results

### Descriptive Statistics

Of the 1339 patients belonging to our cohort,^7^ 1128 patients (mean [SD] age, 32.1 [8.3] years; 781 female individuals [69.2%]; 347 male individuals [30.8%]) met the inclusion criteria for this study (Figure 1). Of all PIRA patients, 86 of 277 (31%) developed early PIRA, and 73 of 144 (51%) developed active PIRA. The median (IQR) time of follow-up was 10.5 years (5.2-17.1) years (Table 1). Patients had a median (IQR) number of visits of 16 (7-36), which made a total of 27 355 EDSS assessments. Of the 1128 study patients, 419 (37%) had at least 1 episode of CDA: 277 of 419 (66%) had at least 1 PIRA event, whereas in the remaining 142 (34%), all their CDA episodes qualified for RAW. In our patients with PIRA, who represented the 25% of all patients with a first demyelinating attack, the first PIRA event occurred at a median (IQR) time of 7.2 (4.6-12.4) years, and 86 of patients (31%) developed PIRA within the first 5 years of the disease.

**Table 1.** Description of All Study Patients With a First Demyelinating Event (N = 1128)

Characteristic	All included patients (N = 1128)	PIRA (n = 277)	No PIRA (n = 851)	*P* value, PIRA vs no PIRA	Early PIRA (n = 86)	Late PIRA (n = 191)	*P* value, early PIRA vs late PIRA	Active PIRA (n = 73)	Nonactive PIRA (n = 71)	*P* value, active vs nonactive PIRA
Age at first attack, mean (SD), y	32.1 (8.3)	33.0 (8.2)	31.8 (8.3)	.05^a^	34.8 (7.9)	32.1 (8.3)	.01^a^	31.1 (8.3)	35.2 (8.3)	.004^a^
Sex, No. (%)										
Male	347 (30.8)	87 (31.4)	260 (30.6)	.85^b^	33 (38.4)	54 (28.3)	.12^b^	29 (39.7)	17 (23.9)	.06^b^
Female	781 (69.2)	190 (68.6)	591 (69.4)		53 (61.6)	137 (71.7)		44 (60.3)	54 (76.1)	
Topography of first attack, patient No. (%)										
Optic nerve	385 (34.1)	91 (32.9)	294 (34.5)	.69^b^	20 (23.3)	71 (37.2)	.14^b^	17/73 (23.3)	29/71 (40.8)	.14^b^
Brainstem	283 (25.1)	74 (26.7)	209 (24.6)		27 (31.4)	47 (24.6)		23/73 (31.5)	15/71 (21.1)	
Spinal cord	333 (29.5)	86 (31.1)	247 (29.0)		29 (33.7)	57 (29.8)		26/73 (35.6)	21/71 (29.6)	
Other	121 (10.7)	26 (9.4)	95 (11.2)		10 (11.6)	16 (8.4)		7/73 (9.6)	6/71 (8.5)	
T2 lesion No. category at first attack, patient No. (%)										
0 Lesions	280/1077 (26.0)	54/268 (20.2)	226/809 (27.9)	.03^b^	15/83 (18.1)	39/185 (21.1)	36^b^	3/72 (4.2)	21/69 (30.4)	<.001^b^
1-3 Lesions	154/1077 (14.3)	34/268 (12.7)	120/809 (14.8)		7 /83 (8.4)	27/185 (14.6)		10/72 (13.9)	10/69 (14.5)	
4-8 Lesions	137/1077 (12.7)	38/268 (14.2)	99/809 (12.2)		11 /83 (13.3)	27/185 (14.6)		10/72 (13.9)	12/69 (17.4)	
≥9 Lesions	506/1077 (47.0)	142/268 (53.0)	364/809 (45.0)		50/83 (60.2)	92/185 (49.7)		49/72 (68.1)	26/69 (37.7)	
≥1 lnfratentorial lesion category at first attack, patient No. (%)	436/836 (52.2)	83/188 (44.2)	353/648 (54.5)	.02^b^	41/70 (58.6)	64/118 (54.2)	.67^b^	39/54 (72.2)	15/49 (30.6)	<.001^b^
Spinal cord lesion category at first attack, patient No. (%)										
0 Lesions	423/661 (64.0)	111/154 (72.1)	312/507 (61.5)	.11^b^	34/57 (59.6)	77/97 (79.4)	.05^b^	20/33 (61.1)	35/47 (74.5)	.10^b^
1 Lesion	120 /661 (18.2)	20/154 (13.0)	100/507 (19.7)		12/57 (21.1)	8/97 (8.2)		4/33 (12.1)	7/47 (14.9)	
2-3 Lesions	55/661 (8.3)	11/154 (7.1)	44/507 (8.7)		6/57 (10.5)	5/97 (5.2)		6/33 (18.2)	1/47 (2.1)	
≥4 Lesions	63/661 (9.5)	12/154 (7.8)	51/507 (10.1)		5/57 (8.8)	7/97 (7.2)		3/33 (9.1)	4/47 (8.5)	
No. of CEL category at first attack, patient No. (%)										
0 Lesions	523/804 (65.1)	124/190 (65.3)	399/614 (65.0)	.99^b^	39/66 (59.1)	85/124 (68.5)	.18^b^	36/59 (61.0)	39/47 (83.0)	.03^b^
1 Lesion	117/804 (14.6)	28/190 (14.7)	89 /614 (14.5)		14/66 (21.2)	14/124 (11.3)		10/59 (16.9)	2/47 (4.3)	
>1 Lesions	164/804 (20.4)	38/190 (20.0)	126/614 (20.5)		13/66 (19.7)	25/124 (20.2)		13/59 (22.0)	6/47 (12.8)	
Presence of OBs at first attack, No. (%)	577/956 (60.4)	158/234 (67.5)	419/722 (58.0)	.01^b^	50/73 (68.5)	108/161 (67.1)	.95^b^	51/63 (81.0)	35/60 (58.3)	.01^b^
Follow-up characteristics										
Follow-up time, median (IQR), y	10.46 (5.2-17.1)	16.95 (11.5-20.9)	8.65 (4.4-14.1)	<.001^c^	13.0 (6.8-18.7)	17.9 (13.2-21.4)	<.001^c^	17.1 (11.8-21.2)	14.1 (8.3-19.7)	.06^c^
Time to first PIRA, median (IQR), y	7.22 (4.6-12.4)	7.22 (4.6-12.4)	NA	NA	3.8 (3.0-4.4)	10.1 (7.0-14.7)	<.001^c^	6.6 (4.4-10.8)	6.9 (3.4-11.9)	.56^c^
ARR considering the whole follow-up, median (IQR)	0.21 (0.1-0.4)	0.17 (0.1-0.3)	0.2 (0.1-0.4)	<.001^c^	0.2 (0.1-0.3)	0.2 (0.1-0.3)	.10^c^	0.2 (0.2-0.4)	0.1 (0.1-0.3)	<.001^c^
No. of patients treated with DMTs at any time during follow-up (%)^d^	580/1109 (52.3)^d^	166/274 (60.6)^d^	414/835 (49.6)^d^	.002^b^	57/84 (67.9)	109/190 (57.4)	.13^b^	64/72 (88.9)	30/71 (42.3)	<.001^b^
Proportion of time receiving DMT during whole follow-up^e^										
Median (IQR)	0.1 (0-0.8)	0.4 (0-0.9)	0 (0-0.8)	.006^c^	0.6 (0-0.9)	0.33 (0-0.8)	.05^c^	0.7 (0.5-0.9)	0 (0-0.7)	<.001^c^
Mean (SD)	0.4 (0.4)	0.4 (0.4)	0.4 (0.4)		0.5 (0.4)	0.4 (0.4)		0.6 (0.3)	0.3 (0.4)	

Abbreviations: ARR, annualized relapse rate; CEL, contrast-enhancing lesion; DMT, disease-modifying therapy; EDSS, Expanded Disability Status Scale; HR, hazard ratio; IT, interaction term; NA, not applicable; OB, oligoclonal band; PIRA, progression independent of relapse activity.

^a^ *t *Test.

^b^ χ^2^ Test.

^c^ Mann-Whitney *U* test (comparison of medians).

^d^ We have excluded those patients who were part of a randomized clinical trial (n = 19) because they could have received placebo (we do not have information on treatment allocation).

^e^ In the numerator, we have only considered those periods of time outside clinical trials, since we are still blinded to treatment allocation in some (n = 51) of those patients who have participated in clinical trials (n = 71), ie, those periods of time within a clinical trial have been considered as if the patient was not treated.

Of those 277 patients who had PIRA, 144 (52.0%) had a recent pre-PIRA MRI done within the 2 years before PIRA. The median (IQR) time between this pre-PIRA MRI and the first PIRA event was 1.0 (0.1-2.0) years. The median (IQR) time between this pre-PIRA MRI scan and the previous MRI (used as reference to assess new T2 lesions in the pre-PIRA scan) was 1.7 (0-10.6) years. Of all 144 patients with recent pre-PIRA MRI information, 73 (51%) developed new T2 lesions before developing PIRA. For patients with PIRA who had (n = 144) and did not have (n = 133) recent pre-PIRA MRI information, MRI and clinical data were similar at baseline (data not shown).

At baseline, patients with PIRA had more T2 lesions in the brain and were more likely to have CSF OB than patients without PIRA (Table 1). Patients with early PIRA were older (mean [SD] age, 34.8 [7.9] years vs 32.1 [8.3] years) and had more spinal cord lesions (≥4 lesions, 5 of 57 [8.8%] vs 7 of 97 [7.2%]) than those with late PIRA. Patients with active PIRA were younger (mean [SD] age, 31.1 [8.3] years vs 35.2 [8.3] years) and more likely to have CSF OBs (51 of 63 [81.0%] vs 35 of 60 [58.3%]) than those with nonactive PIRA and had more brain T2 lesions (≥9 lesions, 49 of 72 [68.1%] vs 26 of 69 [37.7%]) (Table 1).

### Risk of PIRA After a First Demyelinating Attack

**Figure 2.**
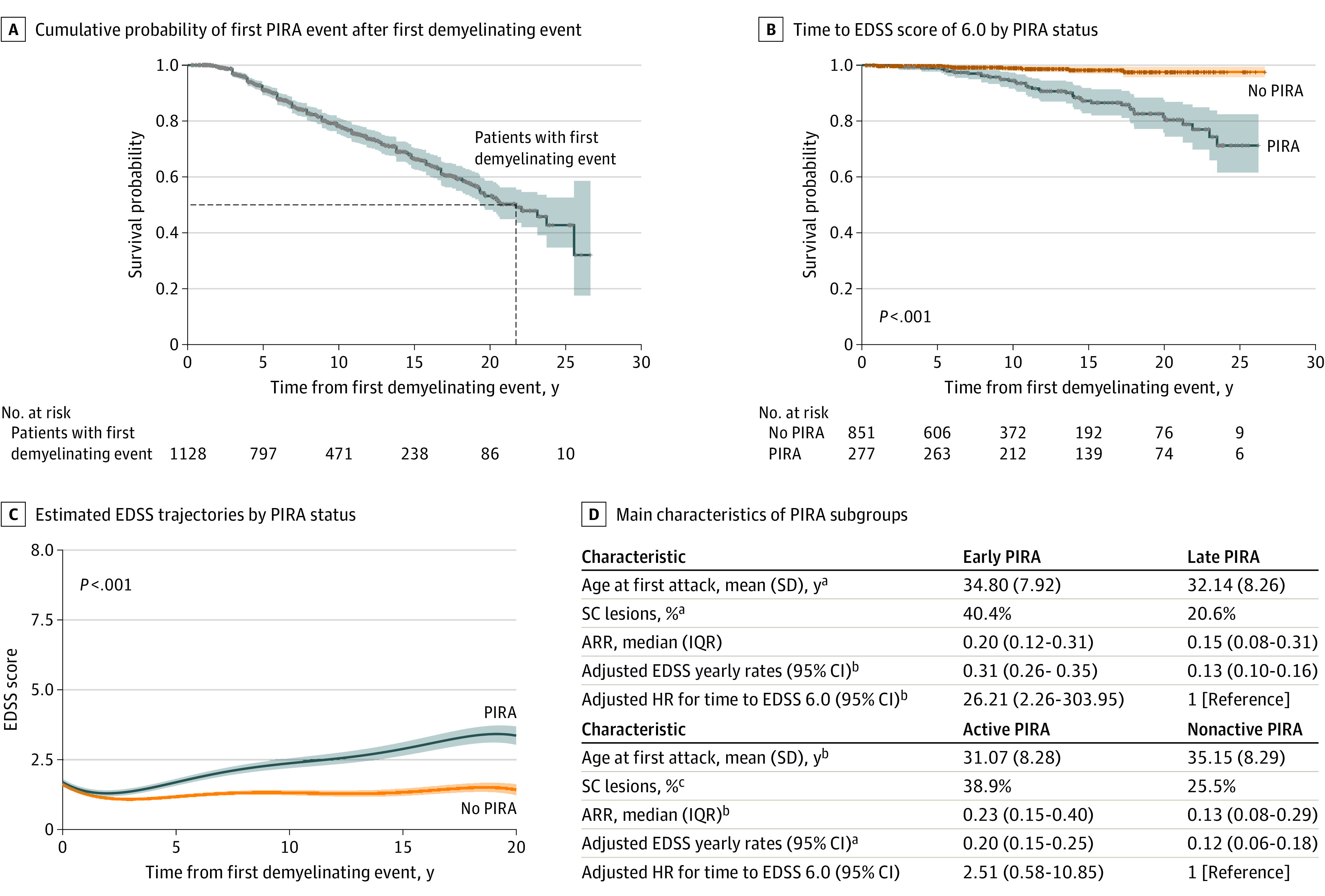
Long-term Outcomes: Progression Independent of Relapse Activity (PIRA) vs No PIRA Comparison and Comparison Between PIRA Subgroups A, Cumulative probability according to Kaplan-Meier estimations of experiencing the first PIRA event after the first demyelinating attack. Our estimations show that approximately 22 years after the first demyelinating event, 50% of all patients will have developed PIRA. B, Cumulative probabilities according to Kaplan-Meier estimations of reaching Expanded Disability Status Scale (EDSS) score of 6.0 in the PIRA and non-PIRA groups. C, Estimated EDSS trajectories over time for patients with and without PIRA. Both panels B and C show that patients with PIRA presented a much worse clinical evolution over time than those without PIRA. D, The main characteristics for the different PIRA subgroups. Early PIRA was associated with steeper EDSS trajectories over time and a higher risk of reaching EDSS 6.0 than late PIRA. Although patients with active PIRA and late PIRA (marginally) differed in terms of yearly EDSS increase rates, no significant differences were observed in survival models after adjusting for confounders. ARR indicates annualized relapse rate; HR, hazard ratio; SC, spinal cord. ^a^*P* < .05. ^b^*P* < .01.

Our Kaplan-Meier analysis revealed that an estimated 8% of all patients (86 of 1128) who present with a first demyelinating attack may develop PIRA within the first 5 years of the disease, and an estimated 50% (564 of 1128) may do so within the first 22 years (Figure 2). Regarding all potential predictors of PIRA at the time of the first attack, only older age was associated with a higher risk of PIRA: for each older decade at first attack, the risk of PIRA increased by 43% (HR for each decade, 1.43; 95% CI, 1.23-1.65; *P* < .001) (eTable 2 in Supplement).

### Long-term Clinical Outcomes

#### PIRA vs non-PIRA

For all patients, the estimated adjusted rate of EDSS increase was 0.07 points per year (95% CI, 0.06-0.09; *P* < .001), although this increase was nonlinear because there was a flattening of the curve as time went along. Patients with PIRA showed a significantly steeper increase in EDSS scores than those without PIRA (Figure 2), which was maintained after adjusting for confounders (0.18; 95% CI, 0.16-0.20 vs 0.04; 95% CI, 0.02-0.05; *P* < .001) (Table 2). Additionally, Kaplan-Meier estimates showed that patients with PIRA had a greater risk of reaching EDSS 6.0 than those without PIRA (Figure 2; Table 2). This was confirmed through adjusted Cox models (HR, 7.93; 95% CI, 2.25-27.96; *P* = .001) (Table 2).

**Table 2.** Prediction of Long-term Outcomes (N = 1128)

Outcome	All study patients (N = 1128)	PIRA (n = 277)	No PIRA (n = 851)	*P* value, PIRA vs no PIRA	Early PIRA (n = 86)	Late PIRA (n = 191)	*P* value, early PIRA vs late PIRA	Active PIRA (n = 73)	Nonactive PIRA (n = 71)	*P* value, active PIRA vs nonactive PIRA
Adjusted yearly EDSS increase rates (95% CI)	0.07 (0.06-0.09)	0.18 (0.16-0.20)	0.04 (0.02-0.05)	<.001	0.31 (0.26-0.35)	0.13 (0.10-0.16)	<.001	0.20 (0.15-0.25)	0.12 (0.06-0.18)	.05
Kaplan-Meier estimates (95% CI) of % patients reaching EDSS 6.0 from the first demyelinating event^a^										
5 y	0.48 (0.06-0.90)	1.09(0-2.31)	0.24 (0-0.57)	<.001	2.41 (0-5.67)	0.52 (0-1.54)	.07	1.37 (0-4.00)	1.52 (0-4.42)	.003
10 y	2.54 (1.41-3.65)	5.58 (2.69-8.39)	1.02 (0.18-1.86)		12.03 (3.71-19.63)	3.24 (0.65-5.76)		9.11 (1.86-15.82)	4.86 (0-10.09)	
15 y	6.00 (3.97-7.98)	12.82 (8.18-17.23)	1.74 (0.42-3.03)		23.93 (11.01-34.98)	9.10 (4.55-3.42)		24.54 (12.18-35.15)	4.86 (0-10.09)	
20 y	9.25 (6.23-12.19)	18.49 (12.37-24.19)	2.45 (0.53-4.33)		23.93 (11.01-34.98)	16.41 (9.47-22.82)		38.32 (22.05-51.19)	10.46 (0-21.50)	
Adjusted HR (95% CI) for reaching confirmed EDSS 6.0 from the first demyelinating event^a^	NA	7.93 (2.25-27.96)	1 [Reference]	.001	26.21 (2.26-303.95)	1 [Reference]	.009	2.51 (0.58-10.85)	1 [Reference]	.22

Abbreviations: EDSS, Expanded Disability Status Scale; HR, hazard ratio; IT, interaction term; NA, not applicable; PIRA, progression independent of relapse activity.

^a^ The Methods section provides full details on model adjustment: outcome EDSS 6.0 was reached when the patient reached that score for the second time; for that analysis, we did not exclude those 10 patients who reached the outcome before the diagnosis of PIRA.

#### PIRA vs RAW

**Figure 3.**
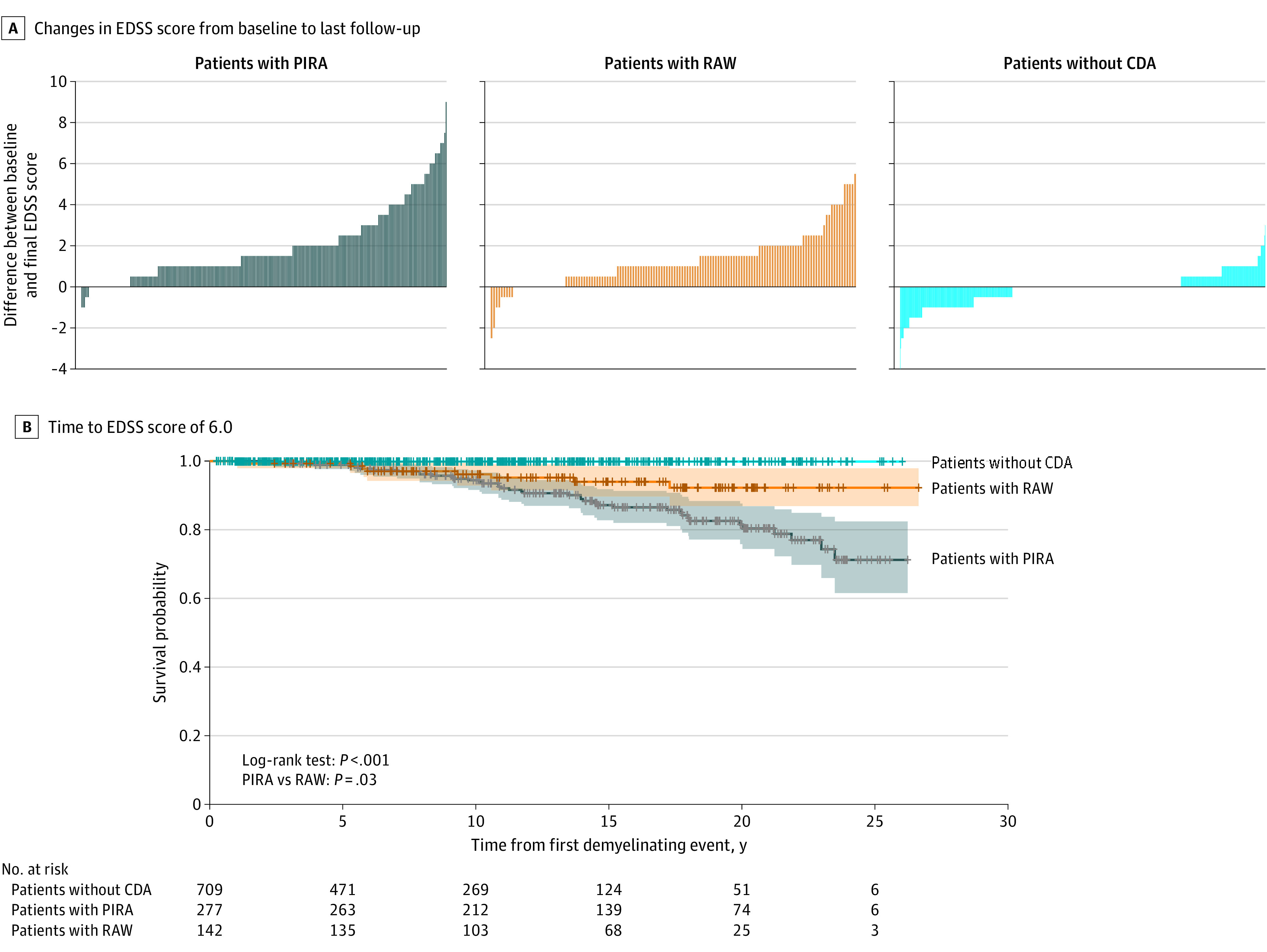
Long-term Outcomes: Progression Independent of Relapse Activity (PIRA) vs Relapse-Associated Worsening (RAW) Comparison A, Crude description of nonconfirmed Expanded Disability Status Scale (EDSS) score changes between baseline EDSS and last clinical visit, for patients with PIRA, RAW, and no confirmed disability accumulation (CDA). Patients with PIRA and RAW seem to be associated with a much heavier disability burden than patients without CDA, even in this unadjusted picture. When comparing PIRA and RAW, PIRA seems to be associated with greater increases in EDSS score than RAW. These findings were then confirmed through survival models of time to EDSS 6.0 (B). B, Kaplan-Meier curves of time to EDSS 6.0 from the first demyelinating attack, for patients with PIRA, RAW, and without CDA. All 3 survival curves were very different (log-rank test, χ^2^_2_ = 44.11; *P *<.001). Patients with PIRA showed significantly faster rates of reaching EDSS 6.0 than patients with RAW (log-rank test, χ^2^_1_ = 4.88; *P *<.03).

No significant differences were observed between patients with PIRA and patients with RAW in terms of yearly EDSS increase rates. However, Kaplan-Meier analyses (Figure 3) and Cox regression models showed that patients with PIRA reached EDSS 6.0 at significantly faster rates than patients with RAW (HR, 4.11; 95% CI, 1.76-9.62; *P* = .001).

#### Early PIRA vs Late PIRA

Patients with early PIRA showed significantly steeper EDSS increase rates than those with late PIRA (0.31; 95% CI, 0.26-0.35 vs 0.13; 95% CI, 0.10-0.16; *P* < .001) (Table 2). Furthermore, patients with early PIRA had a greater risk of reaching EDSS 6.0 at faster rates than those with late PIRA, according to Kaplan-Meier analyses and especially multivariable Cox regression models (HR, 26.21; 95% CI, 2.26-303.95; *P* = .009) (Table 2).

#### Active PIRA vs Nonactive PIRA

Although (unadjusted) Kaplan-Meier analyses showed faster rates of reaching EDSS 6.0 among patients with active PIRA, no significant differences were observed between active PIRA and nonactive PIRA in terms of confounder-adjusted EDSS yearly increase rates or Cox regression models (Table 2). All analyses were repeated using a subcohort of patients who developed 2017 McDonald MS at some time during the follow-up (n = 754), and the results were very similar, being the main messages maintained (eTables 2, 3, 4, and 5 in the Supplement).

## Discussion

In this cohort study, results suggest that one-fourth of all patients presenting with a first demyelinating event may develop a first PIRA event within the first 12 years after symptom onset, and almost 10% may do so within the first 5 years. Further, results suggest that PIRA was associated with, for most patients, a sustained accumulation of disability, which is strongly associated with unfavorable long-term outcomes. In addition, presenting PIRA early in the disease course was associated with an even worse prognosis, independent of the inflammatory burden at the time of the first demyelinating attack.

Our data are similar to those reported by Portaccio et al^6^ in their article investigating PIRA in early MS. Thus, it appears that a substantial proportion of patients with MS may develop progression in the absence of relapses very early in the disease course. This is more typical of a progressive phenotype than of a relapsing-remitting one.^17,18^ Although secondary progressive MS has been classically associated with a minimum level of disability,^19^ apparently a percentage of patients with relapsing MS can become progressive early in the disease course. We believe that these patients should be considered to be patients with progressive MS, with or without MRI inflammatory activity, independent of their disability score or their disease duration. This may have therapeutic implications.

At the time of the first demyelinating event, patients with PIRA were older, slightly more prone to having brain lesions, and more prone to having CSF OBs than those without PIRA. However, despite these differences, predicting which patients would finally develop PIRA based only on baseline characteristics was challenging. Among all clinical and MRI predictors at the time of the first attack, only older age was associated with a greater risk of PIRA in the survival models, in line with previous studies.^2,6^ Interestingly, in the study by Portaccio and colleagues,^6^ apart from older age at study baseline, PIRA could be predicted by the presence of a relapsing-remitting course, a longer disease duration, and a lower number of relapses before the PIRA event.^6^ However, none of these predictors, except for older age, was immediately available at the time of the first attack.

Importantly, patients with PIRA and those without PIRA behaved very differently over time: patients with PIRA showed much steeper EDSS increase rates than those without PIRA and presented an almost 8-fold higher risk of reaching EDSS 6.0 from the first demyelinating event. When those without PIRA were split into RAW and no CDA groups, we observed that patients with RAW and PIRA were clearly different from those without CDA, as expected and reported by previous authors.^6^ Furthermore, patients with PIRA showed a 4-fold higher risk of reaching EDSS 6.0 than patients with RAW. All these findings indicate that early identification of those patients who will develop PIRA may be crucial for managing patients’ expectations and, possibly, for choosing the most appropriate treatment options.

Our analyses revealed that one-third of all patients with PIRA had their first PIRA event within the first 5 years after symptom onset. These patients with early PIRA showed steeper EDSS increase rates and reached EDSS 6.0 much faster than those with late PIRA, even after adjusting for confounders. This suggests that the worse prognosis of early PIRA may be, at least partly, independent of the inflammatory burden and older age at the first attack. This also indicates that more research is needed not only to detect as soon as possible all who will develop PIRA but also to understand the mechanisms leading to PIRA and especially the association between age and early PIRA.

Among those patients with PIRA with recent MRI information before the first PIRA event, one-half of them had their first PIRA in the presence of recent MRI inflammatory activity. Although there was some evidence suggesting that active PIRA might have worse long-term outcomes, this was not confirmed in adjusted models and further research is warranted.

Importantly, we conducted all our analyses on a cohort of patients with a first demyelinating event, regardless of whether they eventually developed MS or not. However, in order to assess to what extent our results could have been affected by the presence of a large proportion of patients who might never develop MS (and therefore never progress), we repeated all our analyses on a subcohort of patients who eventually fulfilled McDonald 2017 MS diagnostic criteria at some point during the follow-up. The results in this subcohort were very similar (almost identical) to those observed in the whole cohort of patients, strengthening the message of our study.

### Limitations

This study has some limitations. One possible limitation stems from the uniquely long follow-up of our cohort and its dynamic nature, which means that patients have been subjected to different diagnostic procedures over time,^9^ which may have potentially affected some of our measurements. For instance, only after 2007 did we begin to perform systematic spinal cord MRI scans at the time of the first demyelinating event. Another consideration refers to the potential effects of treatment. In this study, we included the proportion of follow-up time during receipt of DMTs as a covariate in the mixed-effects models, and a binary time-dependent covariate in the Cox regression models. Additionally, we adjusted all our models for the proportion of follow-up time while receiving high-efficacy DMTs. However, other more complex models might have given slightly different results. This is particularly relevant if we consider that our PIRA group was more exposed to DMTs than the non-PIRA group, which may have meant lower annualized relapse rates and, consequently, higher probability to detect PIRA (because of the longer PFRs). Future studies in this regard are therefore warranted. A further consideration stems from the fact that patients underwent MRIs not only as per protocol but also because they had symptoms or were about to change treatment, which may have altered the observed proportion of patients with active PIRA.

## Conclusions

Results of this cohort study suggest that PIRA is essentially a nonreversible phenomenon associated with unfavorable long-term disability outcomes, especially if such PIRA events occur early in the disease course. Identifying all who will develop PIRA as soon as possible after the first demyelinating event, especially early PIRA, may lead to better treatment choices, and subsequently, better long-term outcomes.

## Notes

Supplement. **eTable 1.** High-Efficacy Disease-Modifying Treatment Distribution of Those Exposed to High-Efficacy Treatments **eTable 2.** Frequency and Percentage Time on High-Efficacy Disease-Modifying Treatment **eTable 3.** Potential Predictors of Time to First PIRA **eTable 4.** Description of Study Patients—Subcohort of Patients Fulfilling McDonald 2017 MS Diagnostic Criteria at Any Time During the Follow-up **eTable 5.** Prediction of Long-term Outcomes—Subcohort of Patients Fulfilling McDonald 2017 MS Diagnostic Criteria at Any Time During the Follow-up
