# Supplementary material for: Association of Early Progression Independent of Relapse Activity With Long-term Disability After a First Demyelinating Event in Multiple Sclerosis
Source: JAMA Neurol. 2022 Dec 19;80(2):151–60. doi: 10.1001/jamaneurol.2022.4655 (PMC9856884; doi:10.1001/jamaneurol.2022.4655)
Supplement: Supplement. — eTable 1. High-Efficacy Disease-Modifying Treatment Distribution of Those Exposed to High-Efficacy Treatments eTable 2. Frequency and Percentage Time on High-Efficacy Disease-Modifying Treatment eTable 3. Potential Predictors of Time to First PIRA eTable 4. Description of Study Patients—Subcohort of Patients Fulfilling McDonald 2017 MS Diagnostic Criteria at Any Time During the Follow-up eTable 5. Prediction of Long-term Outcomes—Subcohort of Patients Fulfilling McDonald 2017 MS Diagnostic Criteria at Any Time During the Follow-up [file jamaneurol-e224655-s001.pdf]

Supplementary Online Content

Tur C, Carbonell-Mirabent P, Cobo-Calvo Á, et al. Association of early progression independent of relapse activity with long-term disability after a first demyelinating event in multiple sclerosis. *JAMA Neurol*. Published online December 19, 2022. doi:10.1001/jamaneurol.2022.4655

eTable 1. High-Efficacy Disease-Modifying Treatment Distribution of Those Exposed to High-Efficacy Treatments

eTable 2. Frequency and Percentage Time on High-Efficacy Disease-Modifying Treatment

eTable 3. Potential Predictors of Time to First PIRA

eTable 4. Description of Study Patients—Subcohort of Patients Fulfilling McDonald 2017 MS Diagnostic Criteria at Any Time During the Follow-up

eTable 5. Prediction of Long-term Outcomes—Subcohort of Patients Fulfilling McDonald 2017 MS Diagnostic Criteria at Any Time During the Follow-up

This supplementary material has been provided by the authors to give readers additional information about their work.

© 2022 Tur C et al. *JAMA Neurology.*

eTable 1. High-Efficacy Disease-Modifying Treatment Distribution of Those Exposed to High-Efficacy Treatments

	**High-efficacy treated patients N = 185**	**PIRA N=70**	**No PIRA N=115**	***PIRA vs no PIRA***	**Early PIRA N=21**	**Late PIRA N=49**	***Early PIRA vs late PIRA***	**Active PIRA N=32**	**Non-active PIRA N=13**	***Active vs non-active PIRA***
**Follow-up characteristics**										
Natalizumab	61 (33%)	23 (32.9%)	38 (33.0%)	p=.99 (∞)	5 (23.8%)	18 (36.7%)	p=.44 (∞)	9 (28.1%)	6 (46.2%)	p=.31 (*)
Rituximab	50 (27%)	29 (41.4%)	21 (18.3%)	**p=.001** (∞)	6 (28.6%)	23 (46.9%)	p=.24 (∞)	14 (43.8%)	7 (53.8%)	p=.78 (∞)
Fingolimod	49 (26.5%)	15 (21.4%)	34 (29.6%)	p=.30 (∞)	6 (28.6%)	9 (18.4%)	p=.36 (*)	5 (15.6%)	2 (15.4%)	-
Ocrelizumab	38 (20.5%)	6 (8.6%)	32 (27.8%)	**p=.003** (∞)	4 (19.0%)	2 (4.1%)	-	4 (12.5%)	-	-
Alemtuzumab	18 (9.7%)	6 (8.6%)	12 (10.4%)	p=.87 (∞)	1 (4.8%)	5 (10.2%)	-	6 (18.8%)	-	-
Cladribine	12 (6.5%)	3 (4.3%)	9 (7.8%)	-	1 (4.8%)	2 (4.1%)	-	1 (3.1%)	1 (7.7%)	-
Mitoxantrone	10 (5.4%)	5 (7.1%)	5 (4.3%)	-	-	5 (10.2%)	-	3 (9.4%)	1 (7.7%)	-
Ofatumumab	6 (3.2%)	3 (4.3%)	3 (2.6%)	-	2 (9.5%)	1 (2.0%)	-	1 (3.1%)	1 (7.7%)	-
Cyclophosphamide	5 (2.7%)	3 (4.3%)	2 (1.7%)	-	1 (4.8%)	2 (4.1%)	-	2 (6.2%)	1 (7.7%)	-

eTable 1 (footnote): (∞): Chi-square test (*) Fisher test due to low frequencies

© 2022 Tur C et al. *JAMA Neurology.*

eTable 2. Frequency and Percentage Time on High-Efficacy Disease-Modifying Treatment

	**All included patients N = 1,109**	**PIRA N=274**	**No PIRA N=835**	***PIRA vs no PIRA***	**Early PIRA N=84**	**Late PIRA N=190**	***Early PIRA vs late PIRA***	**Active PIRA N=72**	**Non-active PIRA N=71**	***Active vs non-active PIRA***
**Follow-up characteristics**										
Number of patients treated with high-efficacy DMTs at any time during follow-up (%)	185 (16.68%)	70 (25.54%)	115 (13.78)	**p=.001** (∞)	21 (25.00%)	49 (25.79%)	p=.99 (∞)	32 (44.44%)	13 (18.31%)	**p=.001** (∞)
Median proportion of time on high-efficacy DMT during whole follow-up (IQR)	0 (0, 0)	0 (0, 0.02)	0 (0, 0)	**p<0.001** (*)	0 (0, 0)	0 (0, 0)	p=.99 (*)	0 (0, 0.16)	0 (0, 0)	**p=.003** (*)
Mean proportion of time on high-efficacy DMT during whole follow-up (SD)	0.06 (0.19)	0.07 (0.18)	0.06 (0.19)		0.08 (0.19)	0.07 (0.17)		0.09 (0.17)	0.08 (0.21)	

eTable 2 (footnote). (∞): Chi-square test; (*): Mann Whitney U test (comparison of medians); For this table, we have considered the whole study cohort with a first demyelinating event not enrolled exclusively in a RCT (N=1,109). *Abbreviations:* DMTs: disease-modifying treatments; HR: hazard ratio; IQR: interquartile range; PIRA: progression independent of relapse activity; SD: standard deviation.

© 2022 Tur C et al. *JAMA Neurology.*

eTable 3. Potential Predictors of Time to First PIRA

Model	Predictor variable	All patients with a first demyelinating attack, N=1,128 HR (95%CI), p-value	PIRA patients among those with a first demyelinating attack, N=277 HR (95%CI), p-value	All patients who fulfill McDonald 2017 MS criteria at any time, N=754 HR (95%CI), p-value	PIRA patients among those who fulfill McDonald 2017 MS criteria at any time, N=209 HR (95%CI), p-value
1	Age at CIS, in decades	**1.43 (1.23; 1.65), p<.001**	**1.24 (1.06; 1.45), p=.007**	**1.32 (1.11; 1.56), p=.001**	**1.43 (1.20; 1.71), p<.001**
2	Male sex	1.06 (0.82; 1.37), p=.67	1.20 (0.92; 1.56), p=.17	1.11 (0.83; 1.48), p=.49	1.20 (0.89; 1.62), p=.24
3	CIS topography, overall p-value	p=.56	p=.60	p=.70	p=.860
	• Optic nerve	1 (ref.)	1 (ref.)	1 (ref.)	1 (ref.)
	• Brainstem	1.01 (0.74; 1.38), p=.95	1.14 (0.83; 1.57), p=.41	1.17 (0.82; 1.67), p=.40	1.01 (0.71; 1.45), p=.95
	• Spinal cord	1.20 (0.89; 1.62), p=.23	1.21 (0.90; 1.63), p=.21	1.18 (0.83; 1.68), p=.37	1.10 (0.77; 1.56), p=.61
	• Other	0.95 (0.61; 1.49), p=.83	1.00 (0.64; 1.57), p=.99	0.96 (0.57; 1.61), p=.87	0.88 (0.52; 1.49), p=.64
4	Brain T2 lesion number category, overall p-value	p=.51	**p=.01**	p=.48	**p=.03**
	• 0 lesions	1 (ref.)	1 (ref.)	1 (ref.)	1 (ref.)
	• 1-3 lesions	0.79 (0.51; 1.22), p=.29	0.96 (0.61; 1.51), p=.87	0.72 (0.35; 1.48), p=.37	0.68 (0.33; 1.43), p=.31
	• 4-8 lesions	1.07 (0.70; 1.65), p=.74	1.36 (0.87; 2.12), p=.18	0.93 (0.46; 1.84), p=.83	1.03 (0.51; 2.07), p=.94
	• ≥9 lesions	1.06 (0.74; 1.51), p=.75	1.67 (1.16; 2.41), p=.006	1.03 (0.55; 1.92), p=.93	1.36 (0.72; 2.56), p=.34
5	Spinal cord lesion number category, overall p-value	p=.74	**p<.001**	p=.69	**p=.002**
	• 0 lesions	1 (ref.)	1 (ref.)	1 (ref.)	1 (ref.)
	• 1 lesions	0.87 (0.54; 1.42), p=.59	2.59 (1.55; 4.31), p<.001	0.77 (0.44; 1.34), p=.36	2.29 (1.28; 4.08), p=.005
	• 2-3 lesions	1.30 (0.68; 2.47), p=.42	2.49 (1.30; 4.78), p=.006	1.22 (0.62; 2.43), p=.57	2.64 (1.32; 5.30), p=.006
	• ≥4 lesions	0.89 (0.47; 1.67), p=.71	1.88 (0.97; 3.62), p=.06	0.93 (0.49; 1.76), p=.82	2.34 (1.22; 4.49), p=.01
6	Number of brain CEL, overall p-value	p=.35	p=.35	p=.30	p=.45
	• 0 lesions	1 (ref.)	1 (ref.)	1 (ref.)	1 (ref.)
	• 1 lesions	0.85 (0.56; 1.29), p=.46	1.35 (0.89; 2.05), p=.16	0.85 (0.55; 1.30), p=.44	1.32 (0.86; 2.03), p=.21
	• >1 lesions	0.76 (0.52; 1.12), p=.16	0.99 (0.67; 1.46), p=.96	0.75 (0.51; 1.09), p=.13	1.02 (0.69; 1.51), p=.91
7	Presence of OBs	1.08 (0.81; 1.45), p=.60	0.93 (0.68; 1.27), p=.64	1.06 (0.73; 1.54), p=.76	0.85 (0.58; 1.25), p=.42

eTable 3 (footnote). Potential predictors after adjusting for DMT exposure, considered as a time-dependent covariate, and adjusting for percentage of time on high-efficacy DMTs. *Abbreviations:* 95%CI: 95% confidence interval; CEL: contrast-enhancing lesions; CIS: clinically isolated syndrome; HR: hazard ratio; OB: oligoclonal bands; PIRA: progression independent of relapse activity.

© 2022 Tur C et al. *JAMA Neurology.*

eTable 4. Description of Study Patients—Subcohort of Patients Fulfilling McDonald 2017 MS Diagnostic Criteria at Any Time During the Follow-up

	**All included patients N=754**	**PIRA N=209**	**No PIRA N=545**	***PIRA vs no PIRA***	**Early PIRA N=67**	**Late PIRA N=142**	***Early PIRA vs late PIRA***	**Active PIRA N=71**	**Non-active PIRA N=40**	***Active vs non-active PIRA***
**Baseline characteristics**										
Age at CIS, mean(SD)	31.62 (8.12)	31.98 (8.04)	31.48 (8.15)	p=.46 (†)	34.00 (8.26)	31.02 (7.79)	**p=.01** (†)	31.18 (8.37)	34.31 (8.21)	**p=.06** (†)
Sex, number of males (%)	232 (30.8)	68 (32.5)	164 (30.1)	p=.57 (∞)	24 (35.8)	44 (31.0)	p=.63 (∞)	27 (38.0)	10 (25.0)	p=.24 (∞)
CIS topography, number of patients (%)				p=.39 (∞)			p=.50 (∞)			p=.83 (∞)
• Optic nerve	213 (28.2)	57 (27.3)	156 (28.7)		15 (22.4)	42 (29.6)		16 (22.5)	12 (30.0)	
• Brainstem	206 (27.3)	66 (31.6)	140 (25.7)		25 (37.3)	41 (28.9)		22 (31.0)	10 (25.0)	
• Spinal cord	252 (33.4)	67 (32.1)	185 (34.0)		20 (29.9)	47 (33.1)		26 (36.6)	14 (35.0)	
• Other	82 (10.9)	19 (9.1)	63 (11.6)		7 (10.4)	12 (8.5)		7 (9.9)	4 (10.0)	
T2 lesion number category at CIS, number of patients (%)				p=.82 (∞)			p=.85 (∞)			p=.28 (∞)
• 0 lesions	42/735 (5.6)	11/206 (5.3)	31/529 (5.9)		3/66 (4.5)	8/140 (5.7)		3/70 (4.3)	5/40 (12.5)	
• 1-3 lesions	94/735 (12.5)	23/206 (11.2)	71/529 (13.4)		6/66 (9.1)	17/140 (12.1)		10/70 (14.3)	3/40 (7.5)	
• 4-8 lesions	117/735 (15.5)	32/206 (15.5)	85/529 (16.1)		8/66 (12.1)	24/140 (17.1)		9/70 (12.9)	7/40 (17.5)	
• ≥9 lesions	482/735 (63.9)	140/206 (68.0)	342/529 (64.7)		49/66 (74.2)	91/140 (65.0)		48/70 (68.6)	25/40 (62.5)	
Infratentorial lesion category at CIS, number of patients with ≥1 lesion (%)	385/591 (65.1)	103/148 (69.6)	282/443 (63.7)	p=.23 (∞)	40/58 (69.0)	63/90 (70.0)	p=.42 (∞)	39/53 (73.6)	14/29 (48.3)	**p=.04** (∞)
Spinal cord lesion category at CIS, number of patients (%)				**p=.009** (∞)			**p=.01** (∞)			p=.32 (∞)
• 0 lesions	224/439 (51.0)	70/108 (64.8)	154/331 (46.5)		20/39 (51.3)	50/69 (72.5)		18/31 (58.1)	14/24 (58.3)	
• 1 lesion	104/439 (23.7)	16/108 (14.8)	88/331 (26.6)		8/39 (20.5)	8/69 (11.6)		4/31 (12.9)	5/24 (20.8)	
• 2-3 lesions	49/439 (11.2)	10/108 (9.3)	39/331 (11.8)		6/39 (15.4)	4/69 (5.8)		6/31 (19.4)	1/24 (4.2)	
• ≥4 lesions	62/439 (14.1)	12/108 (11.1)	50/331 (15.1)		5/39 (12.8)	7/69 (10.1)		3/31 (9.7)	4/24 (16.7)	
Number of CEL category at CIS, number of patients (%)				p=.730 (∞)			p=.62 (∞)			p=.57 (∞)
• 0 lesions	349/620 (56.3)	93/158 (58.9)	256/462 (55.4)		31/57 (54.4)	62/101 (61.4)		36/58 (62.1)	19/27 (70.4)	
• 1 lesion	109/620 (17.6)	27/158 (17.1)	82/462 (17.7)		13/57 (22.8)	14/101 (13.9)		9/58 (15.5)	2/27 (7.4)	
• >1 lesions	162/620 (26.1)	38/158 (24.1)	124/462 (26.8)		13/57 (22.8)	25/101 (24.8)		13/58 (22.4)	6/27 (22.2)	

© 2022 Tur C et al. *JAMA Neurology.*

Presence of OBs at CIS, number (%)	507/658 (77)	142/178 (79.8)	365/480 (76.0)	p=.364 (∞)	45/57 (78.9)	97/121 (80.2)	p=.59 (∞)	49/61 (80.3)	28/37 (75.7)	p=.77 (∞)
**Follow-up characteristics**										
Median follow-up time, in years (IQR)	12.53 (6.70; 18.29)	17.64 (12.36; 21.39)	10.59 (5.51; 16.33)	**p<.001 (*)**	13.28 (9.20; 18.88)	19.00 (14.3; 22.05)	**p<.001 (*)**	17.24 (12.30; 21.28)	15.18 (9.01; 20.54)	p=.17 (*)
Median time to first PIRA (if PIRA), in years (IQR)	7.22 (4.43; 12.60)	7.22 (4.43; 12.60)	NA	NA	3.84 (2.96;4.93)	10.30 (7.14; 14.74)	**p<.001 (*)**	6.57 (4.67; 10.86)	7.02 (3.26; 12.66)	p=.73 (*)
Median ARR considering the whole follow-up (IQR)	0.26 (0.14; 0.43)	0.21 (0.13; 0.38)	0.28 (0.15; 0.44)	**p=.002 (*)**	0.22 (0.13; 0.32)	0.21 (0.12; 0.40)	p=.91 (*)	0.23 (0.16; 0.41)	0.21 (0.13; 0.39)	p=.42 (*)
Number of patients treated with DMTs at any time during follow-up (%) (£)	564/736[£] (76.63%)	162/206[£] (78.64%)	402/530[£] (75.85%)	p=.480 (∞)	54/65 (83.08%)	108/141 (76.60%)	p=.38 (∞)	63/70 (90.0%)	28/40 (70.0%)	**p=.02** (∞)
Median proportion of time on DMT during whole follow-up(IQR) (Ψ)	0.07 (0; 0.54)	0.29 (0; 0.63)	0 (0; 0.47)	**p<.001 (*)**	0.28 (0; 0.65)	0.31 (0; 0.63)	p=.76 (*)	0.53 (0.11; 0.71)	0.09 (0; 0.32)	**p<.001 (*)**
Mean proportion of time on DMT during whole follow-up (SD) (Ψ)	0.26 (0.32)	0.34 (0.33)	0.24 (0.31)		0.32 (0.33)	0.35 (0.33)		0.45 (0.32)	0.20 (0.26)	

eTable 4 (footnote). (†): t-test; (∞): Chi-square test; (*): Mann Whitney U test (comparison of medians); (∞): Chi-square test; (£): we have excluded those patients who have only been treated within a RCT (N=18) because they could have received placebo (we do not have information on treatment allocation); (Ψ): in the numerator, we have only considered those periods of time outside clinical trials, since we are still blinded to treatment allocation in those (N=67) who have participated in clinical trials (i.e. those periods of time within a clinical trial have been considered as if the patient was not treated). *Abbreviations*: DMT: disease modifying therapy; EDSS: expanded disability status scale; HR: hazard ratio; IQR: interquartile range; IT: interaction term; PIRA: progression independent of relapse activity.

© 2022 Tur C et al. *JAMA Neurology.*

eTable 5. Prediction of Long-term Outcomes—Subcohort of Patients Fulfilling McDonald 2017 MS Diagnostic Criteria at Any Time During the Follow-up

	**All study patients N=754**	**PIRA N=209**	**No PIRA N=545**	***PIRA vs no PIRA***	**Early-PIRA N=67**	**Late-PIRA N=142**	***Early-PIRA vs late-PIRA***	**Active-PIRA N=71**	**Non-active-PIRA N=40**	***Active-PIRA vs non-active-PIRA***
**Adjusted yearly EDSS increase rates (95%CI), p-value**	0.09 (0.07; 0.10), p<.001	0.19 (0.17; 0.21), p<.001	0.05 (0.03; 0.06), p<.001	**p<.001**	0.32 (0.27; 0.37), p<.001	0.14 (0.10; 0.17), p<.001	**p<.001**	0.20 (0.15; 0.25), p<.001	0.13 (0.06; 0.21), p=.001	p=.145
**Kaplan-Meier estimates (95%CI) of % patients reaching EDSS 6.0 from the CIS (€), at:**				**p<.001**			p=.09			p=.07
5 years	0.70 (0.09; 1.30)	1.44 (0.00; 3.04)	0.37 (0.00; 0.88)		3.03 (0.00; 7.08)	0.70 (0.00; 2.07)		1.41 (0.00; 4.11)	2.63 (0.00; 7.59)	
10 years	3.43 (1.92; 4.91)	7.17 (3.47; 10.72)	1.43 (0.26; 2.59)		14.27 (4.48; 23.05)	4.31 (0.88; 7.62)		9.14 (1.87; 15.86)	8.45 (0.00; 17.17)	
15 years	7.66 (5.13; 10.13)	16.20 (10.42; 21.60)	2.31 (0.61; 3.97)		28.56 (13.27; 41.15)	11.77 (5.95; 17.24)		24.49 (12.18; 35.08)	8.45 (0.00; 17.17)	
20 years	11.59 (7.89; 15.15)	22.89 (15.51; 29.61)	3.15 (0.78; 5.47)		28.56 (13.27; 41.15)	20.38 (12.00; 27.96)		38.28 (22.03; 51.15)	16.77 (0.00; 32.67)	
**Adjusted HR (95%CI) for reaching confirmed EDSS 6.0 from the CIS (€)**	-	8.63 (2.34; 31.84)	1 (ref)	**p=.001**	20.62 (1.71; 248.17)	1 (ref)	**p=.02**	2.55 (0.58; 11.09)	1 (ref)	p=.21

eTable 5 (footnote). Please see the methods section for full details on model adjustment; (€): outcome EDSS 6.0 was reached when the patient reached that score for the second time; for that analysis, we did not exclude those 10 patients who reached the outcome before the diagnosis of PIRA, to avoid an unbalanced benignisation of the groups being compared. *Abbreviations*: EDSS: expanded disability status scale; HR: hazard ratio; IQR: interquartile range; IT: interaction term; PIRA: progression independent of relapse activity.

© 2022 Tur C et al. *JAMA Neurology.*
